# *Plantago Ovata* Husk: An Investigation of Raw Aqueous Extracts. Osmotic, Hydrodynamic and Complex Rheological Characterisation

**DOI:** 10.3390/molecules28041660

**Published:** 2023-02-09

**Authors:** Kacper Kaczmarczyk, Joanna Kruk, Paweł Ptaszek, Anna Ptaszek

**Affiliations:** 1 Department of Engineering and Machinery in Food Industry, Faculty of Food Technology, University of Agriculture in Kraków, ul. Balicka 122, 30-149 Kraków, Poland; 2 Department of Fermentation Technology and Microbiology, Faculty of Food Technology, University of Agriculture in Kraków, ul. Balicka 122, 30-149 Kraków, Poland

**Keywords:** *Plantago ovata*, psyllium husk, mucilage, raw extract, extensional flow, extensional viscosity, normal force, overlap concentration, relaxation times, osmotic properties

## Abstract

The aim of the study was to characterize raw aqueous extracts from *Plantago ovata* husk in terms of molecular chain mass, osmotic, hydrodynamic, and rheological properties. The raw extracts used in this study have not been yet investigated in the indicated research area. Determination of the molecular weight of the chains present in the extract was performed by gel permeation chromatography (GPC). Osmotic properties were characterized using membrane osmometry. Rheological properties were investigated via classical rotational rheology with normal force measurements, as well as less common but equally important measurements of extensional viscosity. Two types of chains with an average molecular mass of 200 and 1780 kDa were found. The values of the first virial coefficient (B_2_) indicate the predominance of biopolymer-biopolymer interactions. The hydrodynamic radius established at 25 and 30 °C was 74 and 67 nm, respectively, and lower than at 40 °C (>600 nm). The first critical concentration was determined: $c^{*}=0.11\;g\cdot {\mathrm{dL}}^{-1}$. The dominance of negative normal force values resulting from the formation of a pseudo-gel structure of the heteroxylates was demonstrated. Extensional viscosity measurement results revealed that the studied extracts cannot be treated as simple shear-thinning fluids, as indicated by shear flow, but should be considered as viscoelastic fluids.

## 1. Introduction

Polysaccharides extracted from plant seeds constitute a broad group of hydrocolloids used in the food industry for their dietary and technological properties. This group of polysaccharides includes: the non-starch polysaccharides (NSPs) present in the seed endosperm (mainly galactomannans), hemicelluloses being part of endosperm cell walls and finally—the polysaccharide fraction (mucilages) found in the husk (seed coating). The growing interest in plant mucilages has its source in their applicative benefits. They participate in the regulation of blood lipid and glucose levels and also delay the feeling of hunger [1]. They are attributed to strong antioxidant properties and high prebiotic potential. The high water absorption capacity of mucilages [2,3], as well as their ability to gel [4,5,6,7], may construct a basis for the production of foods or dietary supplements that increase a feeling of satiety. From a technological point of view, the mentioned features make them valuable food additives, shaping the rheological properties of products, stabilizing multiphase gas–liquid, liquid–liquid systems, and ingredients preventing syneresis through their ability to permanently retain water in the structure of the food product. So far, the most-studied plant mucilages are polysaccharides derived from sage seeds, basil, chia, *Plantago ovata*, charlock and flax. The best-understood properties of mucilages are derived from cereal seeds [8,9,10,11].

The functional component of whole psyllium seeds is the polysaccharide fraction (mucilage), which can be extracted from both the husk and seed. The polysaccharide that forms the mucilage is a heteroxylan consisting mainly of (1 → 4) linked β-D-xylose with side chains at positions C-3 or C-2, containing arabinose (A) and xylose (X) in various proportions [4,5,7]. In addition, small amounts of rhamnose and galacturonic acid may be present in this fraction, and for this reason, it has been suggested in some studies that the heteroxylanes present in the seed husk exhibit properties analogous to polyelectrolytes and show gelling ability due to the presence of charged acid residues [4,5,6,7].

The properties of the polysaccharide fraction depend on whether it is extracted from the husk [3,5,12,13,14,15] or seeds [2,7,14,16,17,18,19], as well as on the extraction method. The extraction process is carried out with water [3,5,7,12,16,18,19] or alkaline solutions [2,4,5] using different raw materials to solvent ratios and temperatures. Multiple extraction steps are also used [5,7] similarly as under increasing temperature conditions to obtain fractions with increasing average molecular mass and A/X ratios [15]. 

The composition of the dry mucilage preparation is dependent on the raw material (seeds/hulls). In the case of seed extraction, the protein content does not exceed 2.0%, while the polysaccharide fraction usually accounts for 80% to 85% of the preparation, including neutral sugars over 60% [14,17,18]. The ratio of arabinose to xylose (A/X) depends on the extraction conditions, including temperature and a number of extraction steps, and varies from 0.23 to 0.58 [7,14,18]. For husk extraction, the protein content is 3% of the dry preparation [5,12,14,15] while the polysaccharide content can exceed 90% [12]. Temperature increases in extraction from 20 °C to 100 °C determine a significant increase in the A/X ratio from 0.298 to 1.979. [15] The raw material and the method of extraction, including temperature, regulate the values of the average molecular mass of the polysaccharide fraction. In the case of extraction from *Plantago* seeds, weight-average molecular mass values range from Mw = 700 kDa, at a particle size dispersion index of PDI = 1.47 [18] 1085 kDa PDI = 1.3 [7], to 2300 kDa at PDI = 1.15 [17]. Extraction from *Plantago ovata* husk can result in a polysaccharide fraction with a weight average molecular mass ranging from 233 kDa to 1081 kDa [20].

The physicochemical and functional properties of mucilages are investigated after rehydration of the dry preparation of this polysaccharide fraction. Among the most commonly determined are hydrodynamic properties, rheological characteristics [6,7,12,16,18], water absorption, and emulsion-forming ability [2,3,19]. In the group of hydrodynamic properties, the average Rh hydrodynamic radius was determined, with values varying from 24.7 nm [18], 75.5 nm [5] to 403 nm [14]. The size of the hydrodynamic radius depends on the raw material from which the extraction is taken and the degree of purification, for example, values ranging from 240 nm to 283 nm were obtained for polysaccharides extracted from seeds, while values between 148.8 nm and 2122 nm were obtained for the fraction extracted from the husk [14]. From the graphs presented in the work by Patel et al., it is possible to state that there is more than one peak existing in the polysaccharide fraction extracted from the seeds, originating from the dominant chain group. In the case of the reconstituted husk extract, three peaks were found with maxima corresponding to 20 nm, 80 nm, and 250 nm [5]. The temperature of the extraction process also influenced the mean hydrodynamic radius: for the process carried out at 25 °C, the mean hydrodynamic radius of the polysaccharide fraction was 35 nm, while for 65 °C, this totaled 42 nm [7]. An overlap concentration value was also determined for the mucilage from *Plantago ovata*, and was 0.3 g·dL^−1^ for the solution of polysaccharides from the husk in KOH [7], while for aqueous solutions of mucilage extracted from the seeds, this equaled 0.32 g·dL^−1^ [18] and 0.5 g·dL^−1^ [17]. Rheological properties of the reconstituted extracts have also been investigated [6,7,12,16,18], which are characterized by viscoelastic properties. Reconstituted extracts from fractions further derived from successive temperature-elevated extraction steps were found to have gel-like properties, with a ‘stronger’ structure emerging at the solution cooling stage. The rheological properties of these systems can be temperature-scaled (shift-factor) from 20 °C to 90 °C [15]. However, as the results of the study indicate, the method of drying the husk extract significantly affects the functional properties of the reconstituted extracts [21]. 

Literature data are available for rheological measurements for *Plantago ovata* water extract solutions [7,16], but none of them are the results for the extensional flow presented. In recent years, there has been a growing awareness regarding the importance of extensional viscosity next to the shear viscosity of food additions in terms of food safety during the swallowing process [22,23]. The results of such measurements may provide valuable indications as to the suitability and safety of the use of a particular food additive. For that reason, in this paper, the authors of the publication have decided to present both the results for shear flow and extensional flow, and then compare them with each other.

There is a lack of reports in the literature on the properties of raw extracts and reference to the properties of solutions reconstituted from dried or freeze-dried preparations. The solubility and water absorption of dry preparations are studied, however, there is no information on the osmotic properties of raw extracts or their hydrodynamic features. Characterization of the viscoelastic and also viscosity properties of reconstituted extracts is widely discussed, without comparison to raw extracts.

The aim of this study is to fill the lack of knowledge on selected properties of raw extracts obtained from *Plantago ovata* seed husks, using measurement techniques such as GPC, membrane osmometry, DLS and rotational rheology, and extensional viscometry. 

A novelty in the area of research on rheological properties is the determination of changes in normal force: F_n_ and $N_{1}$—the first normal stress difference—as a function of shear rate. In the paper, the results of extensional viscosity measurements are additionally presented as a function of mucilage concentration in the raw extract and elongation rate. Furthermore, in this paper, an attempt is made to correlate hydrodynamic and osmotic properties at $c<c^{*}$ with rheological properties, including changes in the normal force generated during shear flow. These findings are then discussed in light of knowledge of the conformation adopted by arabinoxylans in aqueous solutions.

## 2. Results

### 2.1. Composition and Molecular Characterisation of the Raw Extract

**Figure 1:**
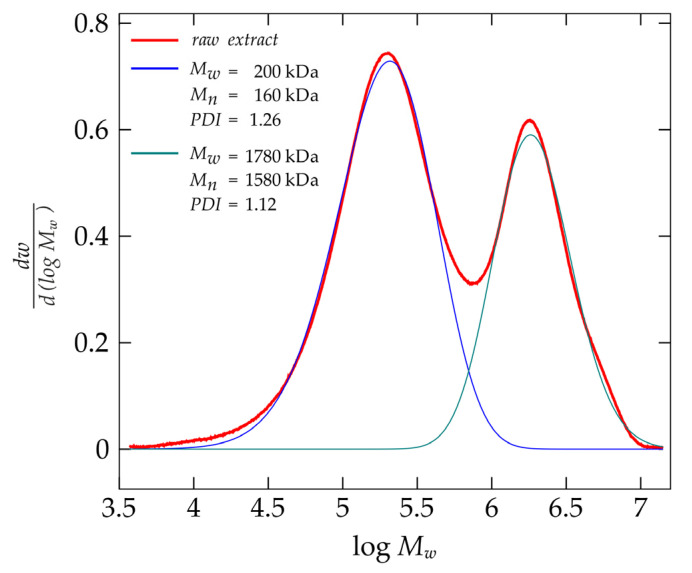
Molecular weight distribution of the polymer chain in the raw *Plantago ovata* extract.

The source material for the raw extract was *Plantago ovata* husk, being predominantly fiber and having a protein content of 1.9%. Extraction was carried out at 25 °C. Under these conditions, the protein content of the extract was 0.9%. The characteristics of the raw extract are shown in the GPC chromatogram. The size distribution of the biopolymer chains is bi-modal, with the global maximum corresponding to a weight-average molecular mass of 200 kDa, while the second maximum was recorded for a value of 1780 kDa. It is worth noting that the intensity of the second maximum is very high, representing as much as 83% of the global peak intensity. Mn, Mw and PDI values for each peak are presented on Figure 1.

### 2.2. Testing the Osmotic and Hydrodynamic Properties of Dilute Solutions

**Figure 2:**
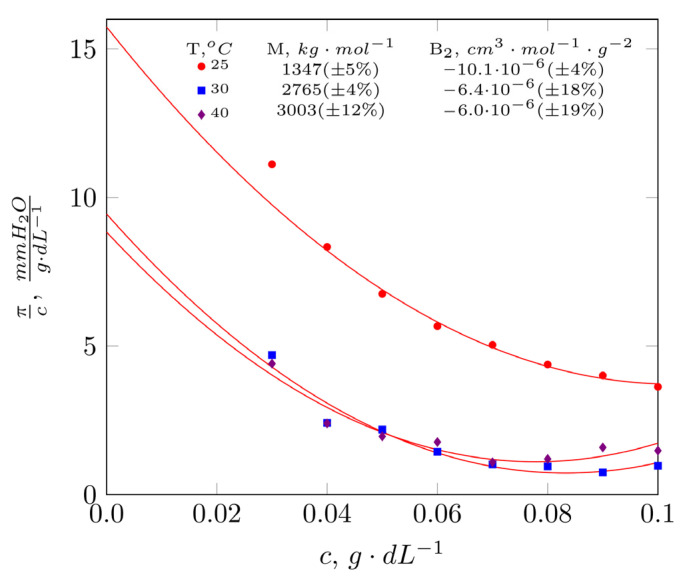
Reduced osmotic pressure as a function of extract concentration at selected temperatures.

**Figure 3:**
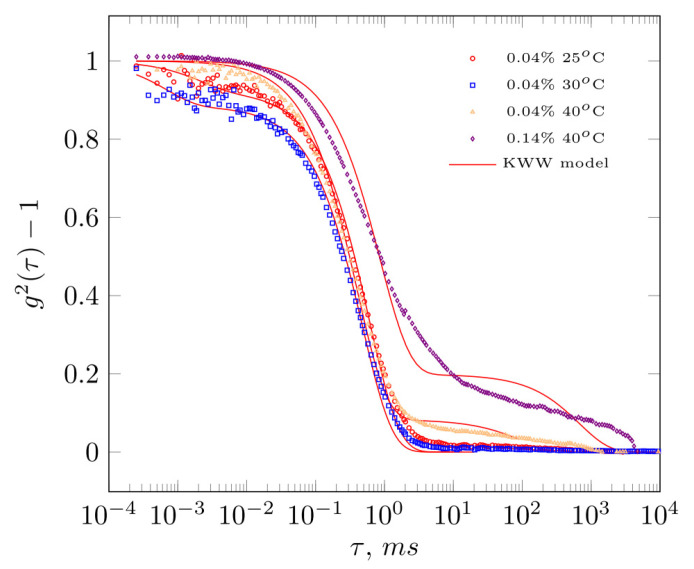
Intensity of autocorrelation function versus delay time for *Plantago ovata* raw husk extract of 0.04 and 0.14 g·dL^−1^ at selected temperatures.

Testing the properties of the solutions in a dilute state (0.03–0.10 g·dL^−1^) included measurement of osmotic pressure and hydrodynamic properties using DLS. The results are presented in Figure 2 and Figure 3 and values of model parameters (Equations (1) and (3)) are shown in Table 1. In the concentration range corresponding to dilute conditions, a non-linear temperature dependence of the reduced osmotic pressure was observed (Figure 2). The values of reduced osmotic pressure decrease with increasing mucilage concentration in solution. The highest $\frac{\pi }{c}\;$values characterize solutions tested at 25 °C. Increasing the temperature to 30 °C and 40 °C results in a decrease in reduced osmotic pressure. The values of the second virial coefficient determined from the experimental data are negative, indicating the predominance of biopolymer-biopolymer interactions. The values of the first virial coefficient—the average osmotic molecular mass—are higher than 1000 kg·mol^−1^ and experience an increase along with a rise in temperature (Table 1). It is noteworthy that at 25 °C, arabinoxylan chains exhibit a limited affinity for water, as manifested by the lowest $B_{2}$ value and average osmotic molecular weight. As temperature increases, $B_{2}$ decreases in absolute value, which results in enhanced biopolymer-water interactions.

Extracts with a concentration of 0.04 g·dL^−1^ at 25, 30 and 40 °C, and 0.14 g·dL^−1^ at 40 °C, were studied using DLS (Figure 3). The course of the autocorrelation function at the different temperatures indicates the existence of one main fraction of polysaccharide chains at 25 °C and 30 °C and two fractions at 40 °C for both of the tested solutions. For this reason, the KWW equation (Equation (3)) was used to describe the relaxation phenomena, the form of which takes the possibility of the hydrodynamic property shaping into account by fast and slow phenomena. As a result, an interesting illustration of arabinoxylan chain behaviour was obtained in the extract. At 25 °C and 30 °C, one relaxation phenomenon dominated—the autocorrelation function decayed in the time interval from 1–2 ms. Consequently, the determined A-values capturing the contribution of the fast relaxation phenomena are small and equal to 8% at 25 °C and 11% at 30 °C, respectively (Table 1). In the case of tests carried out at 40 °C, for both the 0.04 g·dL^−1^ and 0.14 g·dL^−1^ solutions, an increase in relaxation phenomena was evident, which decayed after approximately 1000 ms. Under these conditions, the proportion of fast relaxation phenomena also increased to 0.92 and 0.80 (Table 1). At lower temperatures (25 and 30 °C), the hydrodynamic radius determined from slow relaxation times (Equation (5)) totaled 74 nm and 67 nm, respectively. At 40 °C, $R_{s}$ values were estimated above 600 nm. The increase in the proportion of slow relaxation phenomena was accompanied by a significant increase in the hydrodynamic radius $R_{f}$ values from 5 to 69 nm (for an extract of 0.04 g·dL^−1^) and 152 nm (0.14 g·dL^−1^).

### 2.3. Determination of the First Critical Concentration $c^{*}$

By comparing the apparent viscosity values recorded under conditions of the highest shear rate (rotational rheometer experiment) for all the studied extracts, it was possible to determine the dependence of reduced viscosity on extract concentration. It was also plausible to establish [η] = 32.60 $g^{-1}\cdot \mathrm{dL}$ and overlap $c^{*}=0.11\;g\cdot {\mathrm{dL}}^{-1}$. The value of Huggins constant was 0.42 < 0.5 and therefore, we are dealing with a good solvent [16].

### 2.4. Extensional Viscosity and Shear Viscosity Comparision of Dilute Solutions

**Figure 4:**
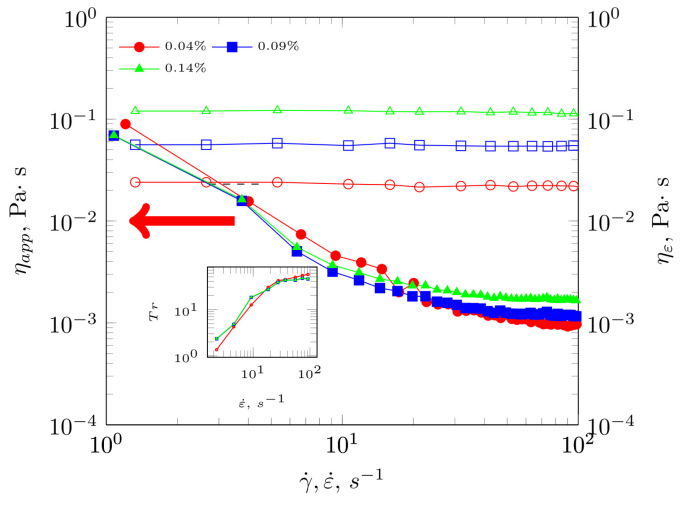
Comparison of changes in apparent viscosity against shear rate and extensional viscosity against extension rate for extracts in dilute regime.

In the range of solution concentrations up to the overlap concentration ($c<c^{*}=0.11\;g\cdot {\mathrm{dL}}^{-1}$), the behaviour of the solutions is characteristic of shear thinning systems, whereby, once the shear rate limit exceeds 40 s^−1^, the systems behave as Newtonian systems with viscosities higher than those of water (Figure 4). For the lowest concentration extracts, no behaviour characteristic for time-dependent fluids was observed. This means that the course of the dependence regarding apparent viscosity on shear rate does not exhibit the existence of a characteristic hysteresis loop. Consequently, there is no energy dissipation during shear flow (Table 2).

In Figure 4, the dependence of extensional viscosity and dynamic apparent viscosity is shown for *Plantago ovata* husk solutions. The dependence of extensional viscosity was practically constant as a function the of extension rate and only an increase in its value was observed as the concentration of the polysaccharide preparation increased. The dependence of apparent viscosity on shear rate demonstrated a shear-thinning effect. Using the $T_{R}$ Trouton ratio, it was found that the value was not constant and was more than 3η above the range of the analyzed shear rates. The fact that this postulate was not fulfilled means that the studied fluid also exhibited elastic properties and cannot be classified as a simple shear-thinning fluid, but as a viscoelastic one.

A qualitative comparison of the shape of the flow curves for extensional viscosity and shear viscosity allowed us to indicate different characteristics of the phenomena. As already mentioned, the behaviour of extensional viscosity as a function of extension rate remained constant, while the behaviour of dynamic viscosity was non-linear. This means that the examined systems underwent a series of transformations during shearing and stretching. The mentioned lack of occurrence concerning a constant value for the Tr number was indicative of the material’s viscoelasticity, but also of its anisotropy, i.e., different mechanical properties depending on the observed direction of stress occurrence. Further analysis of these phenomena showed that they intensified with increasing polysaccharide concentration. This was related to exceeding the individual critical concentrations of the polymer in the solution, which caused structural transformations and intensified the anisotropy of the medium.

### 2.5. Rheological Properties of Raw Extracts in the Semi-Dilute Regime

**Figure 5:**
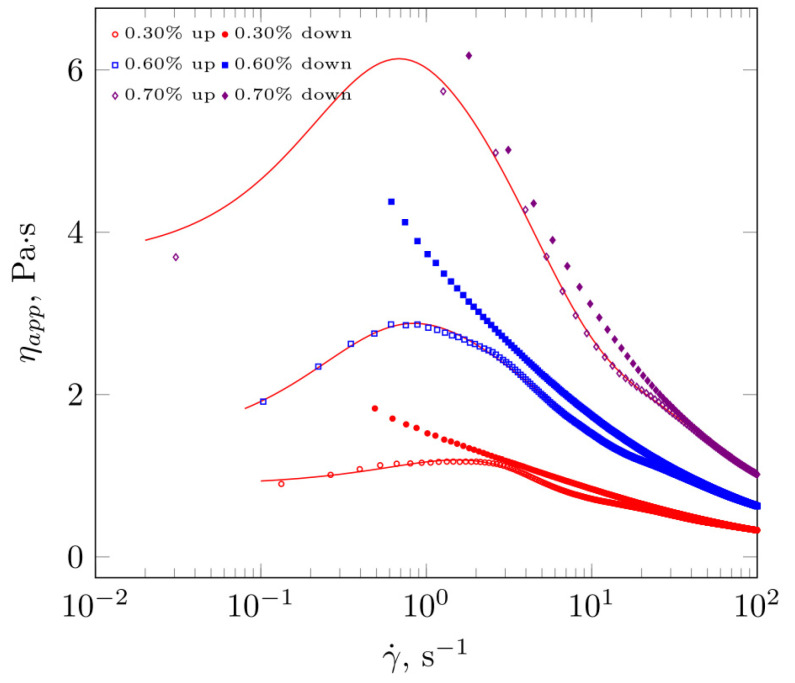
Apparent viscosity dependence on shear rate for increasing (up) and decreasing (down) shear rate values regarding *Plantago ovata* husk extracts.

Deviations from Newton’s law were also noted for extracts near or above the critical concentration. The studied systems behave as shear-thinning fluids with evident time-dependent properties. In the case of extracts with a concentration close to $c^{*}$, the phenomenon of hysteresis as also revealed—the mismatch in the shear rate dependence of apparent viscosity for both stages of the rheological experiment is clearly visible. The differences in eta app values for the same shear rates are of the order of two decades, with the viscosity of the extracts under decreasing shear rate conditions being much higher than at the beginning of the experiment. The viscosity of extracts with $c>c^{*}$ was very high, exceeding 1 Pas. Hysteresis regarding the course of the $\eta_{app}(\dot{\gamma )}$ relationship was not clearly visible for this scale of the figure. Due to this, details concerning the apparent viscosity dependence on shear rate for three extracts with different concentrations higher than the overlapping concentration are shown in Figure 5. At the shear rate range up to about 5 s^−1^, a maximum appears in the apparent viscosity versus the shear rate dependence curve. Once the critical shear rate is exceeded, the apparent viscosity decreases. As the measurements of the rheological properties were carried out according to the hysteresis loop experiment, the $\eta \left(\dot{\gamma }\right)$ curve for this part of the experiment is also shown in the graph. The values of apparent viscosity were significantly higher than those determined in the first part of the experiment, which clearly indicates the phenomenon of mechanical energy storage in the structure of the extracts. In addition, the course of change in apparent viscosity ‘down’ relates to the power model.

The dependence of the apparent viscosity of the extract on the shear rate in the first examination of the rheological experiment was supplemented as a way of modifying the DeKee model (Equation (8)) for the shear-thinning units and the Papanastasiou equation for the shear-thickened ones, and the values of the rheological characteristic times were determined [24]. The results of Equation (8) parameter estimation are given in Table 2. For extracts with $c<c^{*}$, the DeKee model with two characteristic times was fitted, while for extracts with $c>c^{*}$, the best fit was obtained for a combination of two De Kee and Papanastasiou models with three rheological characteristic times.

Rheological characteristic times from the modified DeKee equation (Equation (8)) describing shear-thinning systems (analogy to relaxation times from Maxwell’s model), while lower values can be interpreted as characteristic of systems capable of storing mechanical energy—the smaller the value of this parameter the closer the behaviour is to elastic. Characteristic times from the Papanastasiou equation (analogous to the retardation times of the Burger model) with large values (>1 s) also describe the elastic behaviour of the examined system. The calculation results presented in Table 2 indicate a large contribution of elastic phenomena in shaping the rheological properties of the studied extracts. In the case of extracts with a concentration of $c<c^{*}$, two rheological characteristic times were determined based on the DeKee model, the values of which were less than unity. The shortest characteristic times are characterized by the highest intensity, which can be interpreted as an observable contribution of the elastic phenomena to shaping rheological properties. The behaviour of extracts with a concentration higher than $c^{*}$ was complex and required the use of a modified model, simultaneously capturing both shear thickening and thinning. As a result, values for three relaxation times were estimated, with times $t_{1}$ and $t_{2}$ derived from the DeKee model and time $t_{3}$ from the component of the model specific to shear-thickening systems. Its values are the largest and are additionally characterized by the highest intensity. Concurrently, time *t*_1_, describing the shear-thinning phenomenon, takes on small values, characteristic of elastic contribution. The simultaneous occurrence of these two variants exposes the significant influence of elastic properties in shaping changes regarding apparent viscosity as a function of shear rate. At the same time, it is worth emphasizing that the used rheological models allowed to describe the course of the $\eta_{app}\left(\dot{\gamma }\right)$ dependence well, as evidenced by the goodness of fit described by the $\chi^{2}$ value.

### 2.6. Normal Force and First Normal Stress Difference

The rheological experiments also included the measurement of normal force generated during the simple shear force of the sample in the rheometer measuring system. On this basis, the first normal stress difference ($N_{1}=\tau_{11}-\tau_{22}$) was determined. This quantity characterizes the formation of normal stresses in the fluid during shear force. For a generalized Newtonian fluid, $N_{1}$ is close to zero, due to the fact that the medium exhibits isotropic properties. For fluids demonstrating large deviations from a Newtonian fluid, such as those viscoelastic, the isotropy of normal stresses is absent and $N_{1}$ takes on non-zero values. Depending on the structural arrangement of the fluid, $N_{1}$ values can be positive or negative.

**Figure 6:**
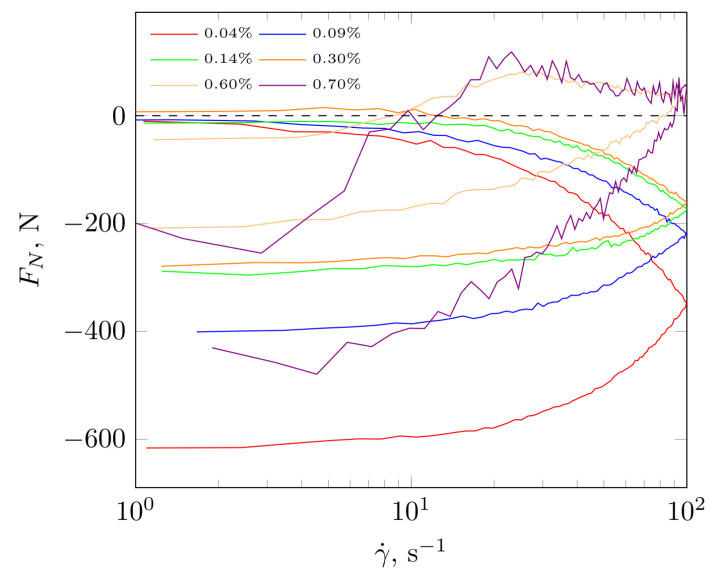
Changes in normal force value as a function of shear rate at increasing and decreasing values.

**Figure 7:**
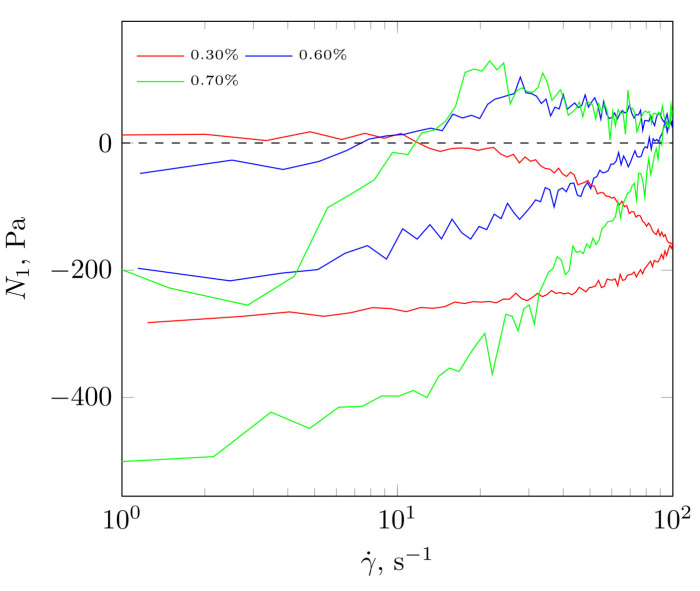
First normal stress difference as a function of shear rate for extracts with the highest concentration at 25 °C.

In Figure 6, the dependence of normal force is presented as a function of the shear rate for diluents with increasing polysaccharide concentration. The lowest concentration was below $c^{*}$ and was characterized by the largest hysteresis. The values of the normal force $F_{n}$ (Figure 6) and the first normal stress difference $N_{1}$ (Figure 7) maintained negative (except for a small area of 5–25 s^−1^). Once $c^{*}$ (higher polysaccharide concentrations) was exceeded, negative values of normal force and first normal stress difference were observed. This meant that the structure formed for low polysaccharide concentrations could not accumulate the energy supplied during shearing and was degraded. This was manifested as a volume contraction, as indicated by the negative normal force and $N_{1}$. There was also no clear refraction in the flow curve indicating the occurrence of shear thickening. Exceeding $c^{*}$ increased the concentration of biopolymer chains in the solution and produced a structure with higher apparent viscosity and the occurrence of a shear thickening region. As the flow of the system was more rigid, certain parts of it could align perpendicular to the current line, causing = expansion of the structure and manifesting itself in positive $F_{n}$ and $N_{1}$ values. 

The experiments were conducted in two phases: increasing and decreasing the value of the shear rate. In the second part of the experiment, when the $\dot{\gamma }$ value was decreasing, the values of the normal force $F_{n}$ (Figure 6) and $N_{1}$ (Figure 7) decreased rapidly. This was due to the fact that the supply of mechanical energy to the system was reduced, which resulted in a breakdown of the formed structure. Furthermore, the flow curve in the case of decreasing $\dot{\gamma }$ was characterized by a simpler structure than in the case of increasing $\dot{\gamma }$. In this situation, it was possible to describe the phenomenon with a power law model. This phenomenon can be explained by the destruction of the fluid-structure described above. Its occurrence was triggered by the lack of sufficient mechanical energy required for its maintenance during the flow. This led to contraction and caused a reduction in the $N_{1}\;$value.

## 3. Discussion

Chromatographic (GPC) analysis resulted in a bi-modal molecular weight distribution, with a global maximum corresponding to 200 kDa and a second, slightly lower intensity, corresponding to 1780 kDa. The weight-average molecular mass corresponded to a value of 200 kDa, which is in agreement with data found in the literature on the subject. Patel et al. [14] obtained a preparation from *Plantago ovata* husk with a weight-average molecular mass ranging from 233 kDa to 1081 kDa. Mucilages extracted from *Plantago ovata* seeds were characterized by slightly higher values: Mw = 700 kDa (Addoun et al. [18]), 1085 kDa (Yu et al. [7]) or 2300 kDa (Benaoun et al. [17]). According to the literature, extraction at 20–25 °C leads to a solution in high yields, rich in a linear fraction with an A/X ratio <0.3 (arabinose to xylose) and therefore, with the lowest degree of substitution [15]. The majority of heteroxylanes present in the *Plantago ovata* husk are poorly soluble in water and form a pseudo-gel structure. These chains most likely form a dendrimeric structure [9], based on interactions between three-fold twisted ribbons [25], forming a weak gel in nature. The essence of the interactions with water is most likely due to the fact that its molecules fill the space between xylan chains and stabilize the whole structure through hydrogen bonds. These factors cause the arabinoxylan chains present in *Plantago ovata* husk to exhibit semi-flexible behaviour [9], similar to very stiff xanthan gum [8,15]. The presence of a second maximum for molecular masses within the order of 1780 kDa and the high proportion of slow relaxation phenomena observed during the DLS experiment may have been caused by the significant proportion of long chains in the population of extracted biopolymers. Furthermore, their presence may explain the low overlap value of $c^{*}=0.11\;g\cdot dL^{-1}$. The $c^{*}$ values presented in the literature range from 0.3 g·dL^−1^ for a solution of the shell polysaccharides in KOH (Yu et al. [7]), 0.32 g·dL^−1^ (Addoun et al. [18]) to 0.5 g·dL^−1^ (Benaoun et al. [17]). It is greatly probable that the conformation adopted in aqueous systems, analysis of the concentration- and temperature-dependence of the reduced osmotic pressure showed negative values of the second virial coefficient B_2_, indicating the predominance of biopolymer-biopolymer interactions. As temperature increases, B_2_ decreases in absolute value, which translates into enhancement of biopolymer- water interactions. The conclusions from the $\;\frac{\pi }{c}$ analysis were confirmed by the results of DLS measurements. This provided an interesting image regarding the behaviour of arabinoxylan chains in the extract. At 25 °C and 30 °C, one relaxation phenomenon dominates, as the proportion of fast relaxation processes is small, 8% at 25 °C and 11% at 30 °C, respectively. When tested at 40 °C, a prolongation of relaxation phenomena is evident, with an increase in the proportion of fast relaxation phenomena to A = 0.92 and A = 0.80 (Table 1). The increase in the proportion of the fast relaxation phenomena was coupled with a significant increase in the value of the hydrodynamic radius R_f_ from 5 nm to 69 nm and 152 nm. At the same time, the osmotic results show an increase in the values of the average osmotic molecular masses. These results can be interpreted as the effect of increased elasticity and, consequently, water absorption of the arabinoxylan chains.

Below $c^{*}$ negative normal force and first normal stress difference values were observed. This behaviour is due to the presence of heteroxylanes, which formed a pseudogel-like, dendritic structure in the extract [9]. This meant that the created structure for low concentrations of polysaccharide $c<c^{*}$ was unable to accumulate the energy supplied during shearing and was degraded. This was manifested as a volume contraction, as indicated by the negative value of the normal force and $N_{1}$. A similar image was obtained from the analysis of the characteristic rheological times, which indicated the presence of elastic contributions to the formation of rheological properties, but an increase in shear rate caused irreversible deformation of the sample and manifested itself as shear thinning. In the case of aqueous polysaccharide solutions, a range of such behaviours is observed, caused by structural transformations occurring during the supply of mechanical energy to the system (shear). Depending on the supplied amount of mechanical energy, the resistance of the fluid structure may be destroyed. Then shear thinning is observed and $N_{1}$ values become negative. From a molecular point of view, this means that the polymer chains in the flow become disentangled and align themselves parallel to the current lines, causing contraction of the entire system and negative $N_{1}$ values are then generated. For structures with high mechanical resistance and a specific structure—making a different scenario possible. For low shear rates, the fluid stores’ energy in its structure, which becomes slightly packed (low contraction) in space. Then, beyond a certain value of shear rate, the structure expands and then the polymer chains or fragments of polymer chains align perpendicular to the current line, which manifests itself as a shear thickening, at which point the fluid experiences expansion and positive $N_{1}$ values are observed. This phenomenon is dependent on polysaccharide concentration and the amount of supplied energy.

## 4. Materials and Methods

### 4.1. Materials

Psyllium husk (*Plantago ovata* husk), country of origin—India, was purchased at the local market (Radgeb Sp. Z o.o., Wrocław, Polska). Composition declared by the supplier in 100 g of the product was the following: 1.93 g of proteins, 1.7 g of carbohydrates, 0.62 g of fat, and 85 g of dietary fibre.

### 4.2. Methods

#### 4.2.1. Molecular Characterization of Husk Extract and Protein Concentration in Raw Extract

Molecular characterization of the husk extract was performed using gel permeation chromatography (GPC). The system consists of two polymer-based columns—Ultrahydrogel-2000 and Ultrahydrogel-500 (Waters, USA) which are connected in a series with an RI detector (Knauer, Germany). An eluent was used in the form of NaNO_3_ and NaN_3_ solution in water, at a concentration of 0.1 mol·L^−1^ and 0.02%, respectively. The eluent flow rate was set to 0.6 mL·min^−1^ and 100 mL of the sample was injected. The extract used as a sample was prepared using 13 mg of husk in 1 mL of water. Pullulan standards (Shodex, Japan) were applied to perform the calibration procedure according to the previously described method [26]. All reagents and chemicals were purchased from Sigma Aldrich.

The protein content in the husk extract was determined via the Kjeldahl method according to the ISO standard (ISO 1871:200 [27]).

#### 4.2.2. Extract Preparation

Distilled water was poured over the husk. The mixture was then shaken for 24 h at 25 °C. After the set time, the samples were centrifuged to separate the husk at 9000 rpm at 25 °C for 5 min. Samples of 0.03; 0.04; 0.05; 0.06; 0.07; 0.08; 0.09; 0.1 g·dL^−1^ were prepared for osmometric measurements. However, for dynamic light scattering (DLS) and rheological measurements, 0.04, 0.07, 0.09, 0.14, 0.30, 0,60, and 0.70 g·dL^−1^ were used.

#### 4.2.3. Osmotic Pressure Measurements

The osmotic pressure measurements of husk extracts were taken with the OSMOMAT 090 membrane osmometer (Gonotec, Berlin, Germany). A two-layer cellulose membrane was applied with a cut-off value of 20,000 Da. Measurements were carried out at three selected temperatures: 25, 30, and 40 °C, to the nearest 0.1 °C. Four repetitions were performed for all testes extracts. The experimental data were used to estimate the osmotic equation of state parameters:

(1) $$\frac{\pi }{c}=\frac{RT}{M_{osm}}\cdot \left[1+B_{2}\left(T\right)\cdot c+B_{3}\left(T\right)\cdot c^{2}+B_{4}\left(T\right)\cdot c^{3}\right]$$

where: π is the osmotic pressure, mmH_2_O, c is the concentration of the dissolved substance, g·dL^−1^, R is the gas constant, T is temperature, $M_{osm}$ is the average osmotic molecular mass, and $B_{2}\left(T\right)$, $B_{3}\left(T\right)$, $B_{4}\left(T\right)$ are the osmotic virial coefficients. Estimation of Equation (1) parameters was carried out using the non-linear algorithm proposed by Marquardt-Levenberg. The target function was formulated as:

(2) $$\chi_{M-L^{|}osm}^{2}=\overset{N}{\underset{j=1}{\sum }}{\left[\frac{\pi_{j}^{\delta }}{c_{j}}-\frac{{\hat{\pi }}_{j}}{c_{j}}\right]}^{2}\underset{M_{osm},\;\;B_{2},\;\;\;B_{3},\;\;\;B_{4}}{\to }\;min$$

where $\frac{\pi_{j}^{\delta }}{c_{j}}$ are values of experimental reduced osmotic pressure and $\frac{{\hat{\pi }}_{j}}{c_{j}}$ were calculated from Equation (1). The minimization procedure estimated the values of the average osmotic molecular mass ($M_{osm}$) and values of osmotic virial coefficient based on experimental data obtained at each single temperature for all extracts (j=1, N).

#### 4.2.4. Dynamic Light Scattering Measurements (DLS)

The raw husk extracts were tested using dynamic light scattering at 25, 30, 40 °C (0.04 g·dL^−1^) and 40 °C (0.14 g·dL^−1^). The measurements were carried out via the Brookheven DLS/SLS system consisting of the BI-160 goniometer with the BI-9000AT digital autocorrelator (Brookhaven, New York, NY, USA). As a source of light, a solid-state laser (JDSU, CDPS532M-050), with an output power of 50 mW at λ = 532 nm was used. The light scattering angle chosen for measurements was 90°. The time average intensity correlation function ($g^{2}\left(\tau \right)-1$) was obtained with the acquisition time of 300 s for each run with the aid of Brookhaven Instruments Dynamic Light Scattering Software version 5.9. The Kohlrausch–Williams–Watts (KWW) stretched exponential function was applied [28]:

(3) $$g^{2}\left(\tau \right)-1\approx {\left\{A\cdot exp\left(\frac{-\tau }{\tau_{f}}\right)+\left(1-A\right)\cdot exp{\left[-\left(\frac{\tau }{\tau_{s}}\right)\right]}^{\beta }\right\}}^{2}$$

where: $g^{2}\left(\tau \right)\;$is the intensity autocorrelation function, $\tau_{f}$, $\tau_{s}$ are relaxation times of the fast and slow components, respectively, β is the exponent of the stretched exponential, τ is the delay time, while A and (1−A) represent the fractional contribution of the two processes. Estimation of parameters (Equation (3)) was performed according to the Levenberg-Marquardt algorithm using the least squares method. The translational diffusion coefficient ($D_{k}$) of fast (*f*) and slow (*s*) components was calculated according to the equation:

(4) $$D_{k}=\frac{1}{t_{k}\cdot q^{2}}\;\;,\;k=f,s$$

where: $q=\frac{4\pi \cdot n}{\lambda }\cdot sin\left(\frac{\theta }{2}\right)$ is s the value of magnitude of the scattering wave vector. The hydrodynamic radius ($R_{h}$) for fast or slow components was calculated based on the Stokes–Einstein equation below [29,30]:

(5) $$R_{h}=\frac{k_{B}\cdot T}{6\pi \cdot \eta_{sol}\cdot D_{k}}\;,\;h=f,s$$

where: $k_{B}$ is the Boltzmann constant, *T* is the absolute temperature on the Kelvin scale, $\eta_{sol}$ is the water viscosity at temperature T.

#### 4.2.5. Rheological Measurements

##### Determination of First Critical Overlap Concentration $c^{*}$

The rheological behaviour of the husk extracts was characterized with the RS6000 rotational rheometer (ThermoFisher, Karlsruhe, Germany) using cone-plate geometry with cone parameters: 60 mm in diameter and 1° Angle. Flow curves were determined at 25 °C, with an increasing and decreasing shear rate of 1 to 100 s^−1^.

The first critical overlap concentration was established on the basis of apparent viscosity values [31] for raw extracts at concentrations of 0.04, 0.07, 0.09, 0.14, 0.30, 0.60 and 0.70 g·dL^−1^ obtained from rotational rheometer measures with a shear rate equal to 100 s^−1^. Calculation of specific viscosity was the first stage of critical overlap concentration determination [32]:

(6) $$\eta_{sp}=\frac{\eta -\eta_{sol}}{\eta_{sol}}$$

where: η is viscosity of the extract and $\eta_{sol}$ is solvent (water) viscosity at 25 °C.

The second stage was reduced viscosity $(\eta_{red}$) calculation, independent of concentrations (c):

(7) $$\eta_{red}=\frac{\eta_{sp}}{c}$$

The subsequent stage was determination of intrinsic viscosity ([η]) according to the equation given below [32]:

(8) $$\eta_{red}=\frac{\eta_{sp}}{c}=\left[\eta \right]\cdot K_{H}\cdot {\left[\eta \right]}^{2}\cdot c$$

where: $\eta_{red}$ is reduced viscosity, $\eta_{sp}$ is specific viscosity, [η] is intrinsic viscosities, c is extract concentration, while *K_H_* is Huggins constant. The estimation of [η] and $K_{H}$ were carried out using the non-linear minimization algorithm proposed by Marquardt–Levenberg. The target function was expressed as follows:

(9) $$\chi_{M-L}^{2}=\overset{N}{\underset{j=1}{\sum }}{\left[\eta_{red}^{\sigma }-{\hat{\eta }}_{red}\right]}^{2}\underset{\left[\eta \right],\;\;\;K_{H}}{\to }\;min$$

where: $\eta_{red}^{\sigma }\;$is the value of experimental reduced viscosity and ${\hat{\eta }}_{red}$ is calculated from Equation (8). The overlap concentration $c^{*}$ was determined from the intersection of the two lines fitted to the experimental data. For this purpose, the plot of viscosity as a function of concentration was used, both quantities being in accordance with the logarithmic scale [33]. 

##### Extensional Rheology

Extensional flow measurements were conducted with the MTS-02 opposite nozzle device (Roman Pomianowski Laboratory of Electronics, Poland), based on the Fuller conception [34], with some alterations. The nozzle’s inner diameter was 2 mm and the distance between nozzles was set to 1 mm. The examined fluids were sucked out by the nozzles through a high-precision double-syringe pump (New Era Pump Systems Inc., USA) at various rates, which translated into different extensional rate values. Measurements were carried out at an ambient temperature of 25 (±0.2) °C at selected elongation rate values within range of 1.3 and 106 s^−1^. The elongation rate was calculated with the following formula:

(10) $$\dot{\varepsilon }=\frac{\frac{\dot{V}}{2}}{A\cdot \frac{d}{2}}$$

where: $\dot{\varepsilon }$—elongation (extension) rate value [s^−1^], $\dot{V}$- volumetric flow rate [m^3^·s^−1^], A–- area of nozzle openings [m^2^], and d—distance between nozzles [m].

Extensional viscosity can be expressed using the formula:

(11) $$\eta_{\varepsilon }=\frac{M}{\dot{\varepsilon }\pi R^{2}d}=\frac{F\frac{d}{2}}{\dot{V}}\;$$

where: $\eta_{\varepsilon }$ is extensional viscosity, Pa·s; M is rotational torque, N·m, $\dot{\varepsilon }$ is elongation (extension) rate value, s^−1^, R is nozzle radius, d—distance between nozzles, m; F is measured force, N; and d is the distance between nozzles, m.

##### Rotational Rheology

The rheological behaviour of the husk extracts was characterised using the RS6000 rotational rheometer (ThermoFisher, Karlsruhe, Germany), with cone-plate geometry having the following cone parameters: 60 mm in diameter and 1° angle. Flow curves were determined at 25 °C, with an increasing and decreasing shear rate of 1 to 100 s^−1^ and 100 to 1 s^−1^, respectively—qualitative hysteresis loop test. The duration of each stage, linear increase (up) and decrease (down) of shear rate, was 750 s. During the experiment, data on changes in the normal force value (F_n_) were also collected. On the basis of the obtained data, the first difference of normal force ($N_{1}$) was determined:

(12) $$N_{1}=\tau_{11}-\tau_{22}$$

where: $N_{1}$ is normal force value, Pa; $\tau_{11},\tau_{22}$ are components of stress tensor, Pa.

The amount of dissipated energy (ΔE, J) was calculated using the area between flow curves obtained during increasing and decreasing shear rate (P, Pa s^−1^), time of experiment (t, s; t = 750 s) and volume of sample (V, m^3^; V = 2,9688·10^−7^). This was performed according to the equation below:

(13) E=P·Δt·V

The obtained rheological data were also applied to estimate characteristic time distributions using the Tikhonov regularisation method. The time constants for shear-thinning and shear-thickening fluids were estimated via rheological equation of the state, which is a combination of the De Kee and Papanastasiou models [35,36]:

(14) $$\eta \left(\dot{\gamma }\right)=\tau_{0}\cdot {\dot{\gamma }}^{-1}+\eta_{1}\cdot \exp\left(-t_{1}\cdot \dot{\gamma }\right)+\eta_{2}\cdot \exp\left(-t_{2}\cdot \dot{\gamma }\right)-\eta_{3}\cdot \exp\left(-t_{3}\cdot \dot{\gamma }\right)$$

In both equations: $\tau_{0}$ is yield stress, Pa; $\dot{\gamma }\;$is shear rate, s^−1^; $\eta_{p}$ is intensity of the time constant parameter, Pa s; and $t_{p}\;$is the time constant, s. Interpretation of parameters was made on the basis of the available study by Ptaszek [37]. Estimation of parameters (Equation (14)) was performed according to the Marquardt–Levenberg method, which was applied as the minimisation algorithm using the least squares method. The target function was defined as follows:

(15) $$\chi_{M-L}^{2}=\overset{N}{\underset{j=1}{\sum }}{\left[\eta_{j}^{\sigma }-{\hat{\eta }}_{j}\left({\dot{\gamma }}_{j}\right)\;\right]}^{2}\underset{\tau_{0},\;\eta_{1},\eta_{2},\;\eta_{3},t_{1},t_{2},t_{3}}{\to }\;min$$

where: $\eta_{j}^{\sigma }$ are experimental values of apparent viscosity and ${\hat{\eta }}_{j}\left({\dot{\gamma }}_{j}\right)$ were calculated from Equation (14).

##### Extensional and Shear Viscosity Comparison—Trouton Ratio

Direct comparison of extensional and shear viscosity can be described by the Trouton ratio, which be defined as follows [38]:

(16) $$T_{R}=\frac{\eta_{\varepsilon }}{\eta_{app}}$$

where: $T_{R}$ is the Trouton ratio or Trouton number, for Newtonian fluids the value is approximately 3, $\eta_{\varepsilon }$ is extensional viscosity and $\eta_{\mathrm{app}}$ is apparent viscosity value determined during shear flow. 

## 5. Conclusions

Two polysaccharide fractions are present in the raw aqueous extracts of *Platago ovata* husk. The predominant one with a weight-average molecular mass of 1780 kDa. Analysis of experimental data from osmometric measurements by using the osmotic equation of state showed that the behaviour of arabinoxylan chains in water is dominated by biopolymer-biopolymer interactions. In contrast, an increase in temperature increases their affinity to water. Dynamic light scattering measurements revealed that at 20 and 30 °C, the proportion of fast relaxation phenomena is low. However, at 40 °C, an increase in the proportion of these phenomena is observed. The application of the modified DeKee and Papanastasiou models revealed the contribution of elastic phenomena in shaping the rheological behaviour of the obtained extracts. The use of apparent viscosity and normal force measurements in combination with extensional viscosity measurements and determination of the Truton ratio allows to comprehensively characterize food liquids, especially polysaccharide extracts. The presented results and their analysis are a valuable contribution to the field of knowledge on polysaccharides due to the use of raw extracts from *Plantago ovata* husk (not subjected to freeze-drying or conventional drying), as data on this type of extract have not yet been presented. 

## Figures and Tables

**Table 1:** Osmotic and hydrodynamic parameters of raw *Plantago ovata* husk extracts.

	Dilute Solutions					Semi-Dilute Solutions		
	$c^{*}=0.11\;g\cdot dL^{-1}$							
T	$M_{osm}$	B_2_ ·10^−6^	A	R_f_	R_s_	A	R_f_	R_s_
°C	kg·mol^−1^	cm^3^·mol^−1^·g^−2^		nm	nm		nm	nm
	0.03–0.10 g·dL^−1^		0.04 g·dL^−1^			0.14 g·dL^−1^		
25	1 347 ± 5%	−10.1 ± 11%	0.08	5	74	-	-	
30	2 765 ± 11%	−6.4 ± 11%	0.11	5	67	-	-	
40	3 003 ± 12%	−6.0 ± 10%	0.92	69	>600	0.80	152	>600

**Table 2:** Values of parameters of the modified DeKee and Papanastasiou model (values with the highest intensity are highlighted) and amount of ΔE dissipated energy.

	Concentration g·dL^−1^	$\tau_{0}$	$t_{1},s$	$t_{2},s$	$t_{3},s$	$\chi^{2}$	ΔE, mJ
dilute	0.04	-	0.15 (1.66%)	0.93 (0.77%)	-	1.81 10^−7^	-
	0.09	-	0.10 (8.25%)	0.60 (1.57%)	-	8.29 10^−8^	-
semi-dilute	0.30	0.31 (0.29%)	0.03 (0.77%)	0.46 (4.60%)	0.71 (5.20%)	2.25 10^−5^	492
	0.60	0.51 (0.49%)	0.02 (0.81%)	0.22 (0.46%)	3.50 (1.05%)	4.03 10^−5^	739
	0.70	0.79 (0.89%)	0.02 (1.18%)	0.24 (0.51%)	3.85 (1.80%)	4.03 10^−4^	970

## Data Availability

The data presented in this study are available on request from the corresponding author. The data are not publicly available due to the adopted research data management plan.
